# Risk of Cancer Among Children and Young Adults With Congenital Heart Disease Compared With Healthy Controls

**DOI:** 10.1001/jamanetworkopen.2019.6762

**Published:** 2019-07-05

**Authors:** Zacharias Mandalenakis, Christina Karazisi, Kristofer Skoglund, Annika Rosengren, Georgios Lappas, Peter Eriksson, Mikael Dellborg

**Affiliations:** 1 Institute of Medicine, Department of Molecular and Clinical Medicine, Sahlgrenska Academy, University of Gothenburg, Gothenburg, Sweden

## Abstract

**Importance:**

Adult patients with congenital heart disease (CHD) have an increased incidence of cancer, presumably owing to repeated radiation exposure, genetic predisposition, or repeated stress factors during heart interventions. However, there are limited data on the risk of cancer in children and young adults with CHD compared with the general population.

**Objective:**

To determine the risk of developing cancer from birth to age 41 years among patients with CHD compared with healthy matched controls.

**Design, Setting, and Participants:**

This registry-based, matched, prospective cohort study in Sweden used data from the Patient and Cause of Death Registers. Successive cohorts of patients with CHD born from 1970 to 1979, 1980 to 1989, and 1990 to 1993 were identified. Each patient (n = 21 982) was matched for birth year, sex, and county with 10 controls without CHD from the general population (n = 219 816). Follow-up and comorbidity data were collected from 1970 until 2011. Data analysis began in September 2018 and concluded in February 2019.

**Main Outcomes and Measures:**

Risk of cancer among children and young adults with CHD and among healthy controls.

**Results:**

Among 21 982 individuals with CHD and 219 816 healthy matched controls, 428 patients with CHD (2.0%) and 2072 controls (0.9%) developed cancer. Among patients with CHD, the mean (SD) age at follow-up was 26.6 (8.4) years, and 11 332 participants (51.6%) were men. Among healthy controls, the mean (SD) age at follow-up was 28.5 (9.1) years, and 113 319 participants (51.6%) were men. By the age of 41 years, 1 of 50 patients with CHD developed cancer. The overall hazard ratio (HR) for cancer was 2.24 (95% CI, 2.01-2.48) in children and young adults with CHD compared with controls. Risk increased by each successive birth cohort to an HR of 3.37 (95% CI, 2.60-4.35) among those born from 1990 to 1993. The risk of cancer was similar in men and women with CHD (men: HR, 2.41; 95% CI, 2.08-2.79; women: HR, 2.08; 95% CI, 1.80-2.41). The HR for cancer among patients with CHD who underwent surgery was 1.95 (95% CI, 1.58-2.33) compared with controls; for patients with CHD who had not undergone surgery, the HR was 2.43 (95% CI, 2.12-2.76). According to a hierarchical classification, a significantly increased risk of cancer was found among patients with complex heart lesions, such as conotruncal defects (HR, 2.29; 95% CI, 1.62-3.25), compared with healthy controls.

**Conclusions and Relevance:**

Children and young adult patients with CHD had an increased risk of developing cancer compared with healthy matched controls, and the risk was significantly higher among patients with CHD from the most recent birth cohort. An increased risk of cancer in all CHD lesion groups was found, and a systematic screening for cancer could be considered for this at-risk group of patients.

## Key Points

**Question:**

What is the risk of developing cancer among children and young adults with and without congenital heart disease?

**Findings:**

In this nationwide registry-based cohort study of 21 982 children and young adults with congenital heart disease and 219 816 healthy matched controls in Sweden, the risk of cancer was more than 2-fold higher among patients with congenital heart disease than among controls.

**Meaning:**

The findings suggest that children and young adults with congenital heart disease have an increased risk of cancer, and a systematic screening for cancer could be considered for this at-risk group of patients.

## Teaser

This cohort study evaluates the risk of developing cancer from birth to age 41 years among patients with congenital heart disease compared with healthy matched controls in Sweden.

## Introduction

Congenital heart disease (CHD) is the most common major congenital disorder, with a prevalence of almost 1% in live births.^1,2^ With the evolution of pediatric care during the last decades, more than 95% of patients with CHD survive into adulthood.^3^ In parallel with increasing survival among patients with CHD, lifetime secondary morbidities might be expected to increase in this group.

Previous studies have shown that the risk of acquired cardiovascular comorbidities is markedly higher among young patients with CHD compared with matched controls from the general population.^4,5,6^ Furthermore, noncardiovascular diseases, and particularly cancer, are associated with CHD. The cause of this association is likely multifactorial, but radiation exposure is probably a factor.^7,8,9,10,11,12^ However, genetic predisposition, socioeconomic status, other comorbidities, and lifestyle factors (eg, a more sedentary lifestyle) are also associated with the development of cancer.^13,14,15,16,17^ In contrast to many other cardiovascular disorders, CHD is present from birth. However, to our knowledge, no study has observed patients with CHD from birth and compared their cancer outcomes with matched controls. Therefore, we aimed to investigate the risk of developing cancer in children and in young adults with CHD compared with matched controls without CHD from birth up to age 41 years.

## Methods

### Study Population and Design

The study population has been described previously.^3,4,6,18^ Through the Swedish Patient Register, we identified patients who were born between January 1970 and December 1993 and were diagnosed as having CHD at any age. Each patient was matched by birth year, sex, and county with 10 controls from the Total Population Register in Sweden. The patients were observed from birth until the occurrence of cancer, death, or the end of the study on December 31, 2011, with a maximum follow-up time of 41 years.

The study was approved by the Gothenburg Regional Research Ethics Board and complied with the Declaration of Helsinki.^19^ In the data set provided by the Swedish National Board of Health and Welfare, every individual’s social security number was replaced with a unique code. Therefore, written informed consent was not required. This report follows the Strengthening the Reporting of Observational Studies in Epidemiology (STROBE) reporting guideline.

### Definitions

Congenital heart disease was defined as present in any patient with at least 1 outpatient visit, hospital discharge, or death certificate with at least 1 registered diagnosis of CHD according to the *International Classification of Diseases, Eighth Revision *(*ICD*-*8*), *ICD-9*, or *ICD-10* (eTable 1 in the Supplement). Cancer was defined by *ICD* codes (eTable 2 in the Supplement). Surgical procedures on the cardiovascular system were classified as codes 30 to 32 in *Classification of Operations*^20^ or F codes in *Classification of Surgical Procedures*.^21^ A hierarchical CHD classification was used to categorize diagnosis of CHD.^22^

### Statistical Analysis

We analyzed the survival of patients and controls born from 1970 to 1993 to determine the association of CHD with the risk of developing cancer. The incidence rates and cause-specific hazard ratios (HRs) for cancer were estimated with 95% CIs to compare patients with CHD with healthy controls who were matched by birth year, sex, and county. Two-sided *P* values were used, and *P* <.05 was considered statistically significant. We used SAS software version 9.4 (SAS Institute) and R software version 3.2 (The R Foundation) to perform all statistical analyses.

## Results

**Figure 1.**
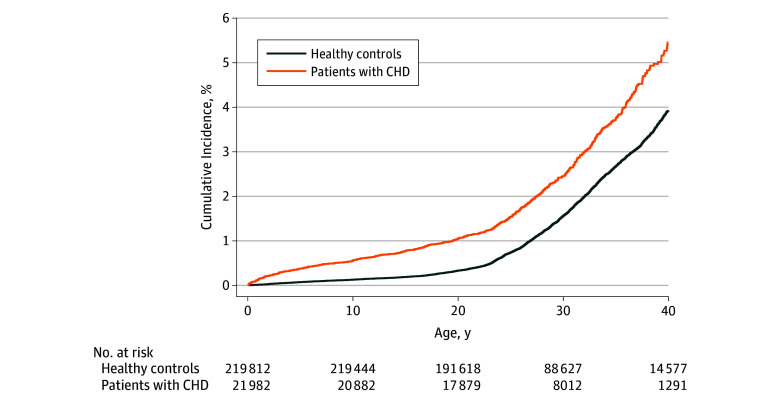
Cumulative Incidence of Cancer in the Study Population CHD indicates congenital heart disease.

We identified 21 982 patients with CHD and 219 816 healthy controls who were matched by birth year, sex, and county. Overall, 11 332 patients with CHD (51.6%) and 113 319 healthy controls (51.6%) were men. The characteristics of the study population are shown in Table 1. Most participants (20 135 patients with CHD [91.6%] and 202 230 healthy controls [91.6%]) were born in Sweden. The mean (SD) age at follow-up was 26.6 (8.4) years (median, 26.2 years; interquartile range, 19.8-32.6 years) for patients with CHD and 28.5 (9.1) years (median 27.5 years; interquartile range, 21.1-33.9 years) for controls. By the age of 41 years, 1 of 50 patients with CHD developed cancer. The cumulative incidence of cancer exponentially increased during adulthood. This increase was higher among patients with CHD than in controls, with up to 4.5% cumulative incidence among patients with CHD and 2.5% cumulative incidence among healthy controls at a maximum age of 41 years (Figure 1).

**Table 1.** Characteristics of the Study Population

Characteristic	No. (%)	
	Patients With CHD	Controls
All participants, No.	21 982	219 816
Men	11 332 (51.6)	113 319 (51.6)
Birth cohort		
1970-1979	7535 (34.3)	75 350 (34.3)
1980-1989	9126 (41.5)	91 266 (41.5)
1990-1993	5321 (24.2)	53 200 (24.2)
Born in Sweden	20 135 (91.6)	202 230 (91.6)
Age at follow-up, y		
Mean (SD)	26.6 (8.4)	28.5 (9.1)
Median (IQR)	26.2 (19.8-32.6)	27.5 (21.1-33.9)

Abbreviations: CHD, congenital heart disease; IQR, interquartile range.

**Figure 2.**
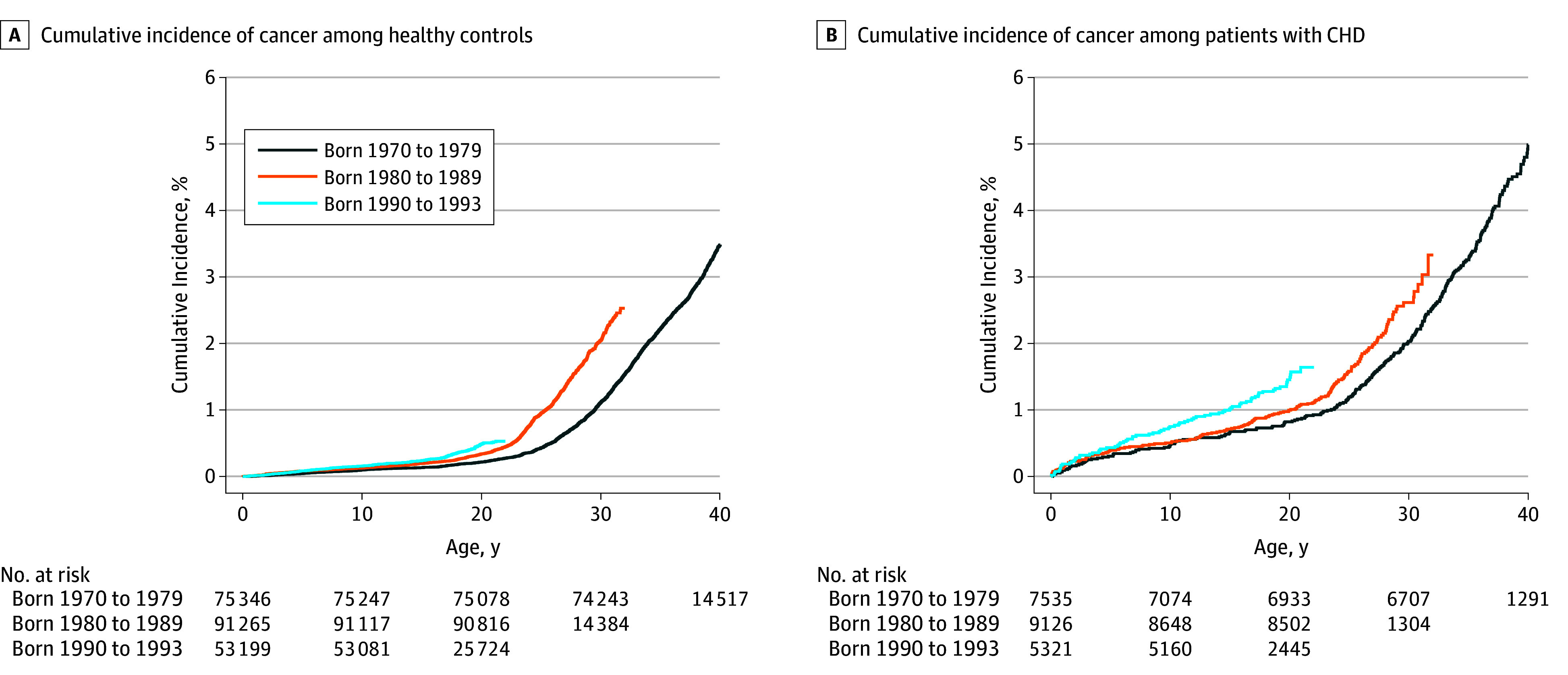
Cumulative Incidence of Cancer in the Study Population by Birth Cohort CHD indicates congenital heart disease.

Table 2 shows the risk of developing cancer in the study population by sex and birth cohort. The overall HR for cancer was 2.24 (95% CI, 2.01-2.48) among patients with CHD compared with healthy controls. The risk of cancer in men and women with CHD was similar, with HRs of 2.41 (95% CI, 2.08-2.79) for men and 2.08 (95% CI, 1.80-2.41) for women. The cumulative incidence of cancer exponentially increased in men and women with CHD until the age of 41 years, with a cumulative incidence of cancer of 4.0% and 5.0%, respectively (eFigure 1 in the Supplement). For comparison of successive birth cohorts, the highest relative risk of developing cancer was found among patients with CHD born from 1990 to 1993 (HR, 3.37; 95% CI, 2.60-4.35). The cumulative incidence of cancer among patients with CHD and in healthy controls exponentially increased by birth cohort. At age 18 years, patients with CHD from the 1990 to 1993 birth cohort had the highest cumulative incidence rate of cancer (1.5%) (Figure 2).

**Table 2.** Risk of Cancer Among Patients With CHD Compared With Matched Controls According to Sex and Birth Period

Characteristic	No. (%)		Hazard Ratio (95% CI)	*P* Value
	Patients With CHD and Cancer	Controls With Cancer		
All patients	428 (1.9)	2072 (0.9)	2.24 (2.01-2.48)	<.001
Men	215 (1.9)	969 (0.9)	2.41 (2.08-2.79)	<.001
Women	213 (2.0)	1103 (1.0)	2.08 (1.80-2.41)	<.001
Birth cohort				
1970-1979	209 (2.8)	1150 (1.5)	2.01 (1.73-2.33)	<.001
1980-1989	142 (1.6)	686 (0.8)	2.21 (1.84-2.64)	<.001
1990-1993	77 (1.4)	236 (0.4)	3.37 (2.60-4.35)	<.001

Abbreviation: CHD, congenital heart disease.

The risk of cancer in different lesion groups was estimated and compared with controls (Table 3). Numerically, the relative risk of cancer was higher among patients with CHD compared with controls for all lesion groups, but one of the highest risks for cancer was found among patients with conotruncal defects (HR, 2.29; 95% CI, 1.62-3.25). However, patients with simple defects, such as coarctation of the aorta (HR, 2.00; 95% CI, 1.27-3.16) or ventricular septal defect (HR, 2.00; 95% CI, 1.57-2.56), had a significantly increased risk of developing cancer. The cumulative incidence of cancer among patients with conotruncal defect, coarctation of the aorta, ventricular septal defect, and other heart and circulatory system anomalies (including isolated congenital valvulopathies) was higher compared with healthy controls (eFigure 2 in the Supplement).

**Table 3.** Risk of Cancer Among Patients With CHD Compared With Matched Controls According to Lesion Group

Lesion Group	No./Total No. (%)		Hazard Ratio (95% CI)	*P* Value
	Cancer in Patients with CHD and Lesion	Cancer in Controls With Lesion		
Conotruncal defects	38/2022 (1.9)	226/20 230 (1.1)	2.29 (1.62-3.25)	<.001
Severe nonconotruncal defects	15/1087 (1.4)	100/10 870 (0.9)	1.94 (1.12-3.35)	.03
Coarctation of the aorta	22/1306 (1.7)	119/13 060 (0.9)	2.00 (1.27-3.16)	.006
Ventricular septal defect	76/4369 (1.7)	402/43 689 (0.9)	2.00 (1.57-2.56)	<.001
Atrial septal defect	44/2405 (1.8)	243/24 049 (1.0)	1.87 (1.36-2.58)	<.001
Other heart and circulatory system anomalies	233/10 793 (2.2)	982/107 918 (0.9)	2.49 (2.16-2.88)	<.001

Abbreviation: CHD, congenital heart disease.

The incidence rates according to the type of cancer diagnosis are shown in eTable 3 in the Supplement. A higher incidence of cancer among patients with CHD was observed in all types of cancer. Some of the most predominant types of cancer among patients with CHD were lymphoma or leukemia and carcinoma.

The incidence rates and rate ratio of cancer among patients with CHD compared with controls by cancer diagnosis are shown in eTable 4 in the Supplement. Malignant neoplasms of the digestive system had the highest incidence rate ratio (3.58; 95% CI, 2.58-4.98). In contrast, the incidence rate ratios of melanoma and other skin cancers were not significantly increased among patients with CHD.

The incidence rates of cancer by age and different lesion groups are shown in eTable 5 in the Supplement. The highest incidence rates were observed during the first 4 years of life and during adulthood, particularly among patients with conotruncal defects and ventricular septal defect.

A total of 8044 patients with CHD (36.6%) underwent at least 1 cardiac surgical procedure before age 41 years. The HR for cancer among patients with CHD who underwent surgery was 1.95 (95% CI, 1.58-2.33), and the HR for patients with CHD who did not undergo surgery was 2.43 (95% CI, 2.12-2.76) compared with healthy controls. We also found that the risk of cancer among patients with surgically corrected ventricular septal defect increased (HR, 2.25; 95% CI, 1.69-3.04) compared with healthy controls. Patients with ventricular septal defect who did not undergo surgery had an HR of 1.93 (95% CI, 1.33-2.90) compared with healthy controls. Furthermore, we found that the HR of benign tumors among patients with CHD was 1.38 (95% CI, 1.32-1.44) compared with healthy controls.

## Discussion

To our knowledge, this is the first study to investigate the long-term incidence of cancer from birth to a maximum age of 41 years among patients with all types of CHD, with or without surgery. We found an increased risk of cancer compared with healthy matched controls that was already present in childhood. The absolute risk of developing cancer among patients with CHD increased for each 10-year birth cohort. Consequently, 1.5% of patients with CHD in the cohort who were born from 1990 to 1993 had developed cancer by age 18 years or younger.

We found a more than 2-fold higher risk among patients with CHD of developing cancer compared with controls, which is marginally higher compared with previous studies.^8,11^ However, the study by Gurvitz et al^11^ only included adult patients with CHD and only compared the prevalence rate of cancer among patients with CHD with that in the general population, not with matched controls. Therefore, these authors had potentially lower precision of estimates. Lee et al^8^ reported a median follow-up of only 5.2 years, whereas follow-up started at birth in our study. We also had a substantially longer follow-up, with median (interquartile range) follow-up of 26.2 (19.8-32.6) years and a mean (SD) follow-up of 26.6 (8.4) years among patients with CHD.

We found that the relative risk of cancer was high, regardless of whether patients had undergone surgical procedures. Our study findings add to the ongoing discussion on whether there is an association of the use of cardiac procedures with the risk of malignancy, potentially mediated by exposure to low-dose ionizing radiation, as suggested by Cohen et al.^7^ However, the radiation dose from cardiac catheterization might not be the only contributor to the increased risk of cancer observed in our study population. Harbron et al^9,23^ reported that the standardized incidence ratio of cancer was 0.90 for children undergoing cardiac catheterizations in childhood when patients who underwent a transplant were censored. Our data would support that the increased risk of cancer in children and young adults with CHD is not a simple function of radiation exposure.

According to the guidelines of the American Cancer Society, a major mechanism for reducing the risk of cancer, and even preventing the occurrence of cancer, is performing physical activity and eating healthy food.^17^ Patients with CHD have lower isotonic muscle function, reduced oxygen uptake, and higher exercise intolerance compared with those without CHD.^24,25^ This further suggests an increased risk of cancer in this group of patients. Furthermore, Keum et al^14^ described the association of physical inactivity with an increased risk of cancer of the digestive system. Interestingly, we found that cancer of the digestive system was the most prevalent among patients with CHD, with an incidence rate ratio of 3.58 compared with any other cancer diagnostic group.

The incidence of cancer continuously increased by birth cohort among patients with CHD and among healthy controls but to a lesser extent between those born from 1980 to 1989 and those born from 1990 to 1993. This observation may reflect a change in the incidence of cancer in general, but it may also reflect an increase in survival. Among patients born from 1970 to 1979, a significant proportion of patients with complex CHD may not have survived long enough to develop cancer.

We previously reported that patients with CHD who were born in the early 1990s showed a high survival into adulthood (96%).^3^ However, in the present analysis, we found that the cumulative incidence of cancer was 1.5% when adulthood was reached. Preventive measures are important for limiting the risk of cancer in the long term. However, with minimizing the risk of cancer in general, particularly among patients with CHD, the value of such measures in young patients is less clear. The importance of limiting radiation dose, potent antibiotics, and general biological stress in young patients with CHD is largely unknown but requires further study. We also found no significant increase in benign tumors among patients with CHD compared with controls, indicating that the overreporting and increased awareness associated with more frequent medical contact is not a likely explanation for our findings.

### Strengths and Limitations

Our study has several strengths, but it also has some limitations. One strength of our study is that follow-up started at birth and included all cancer forms. This minimized selection bias compared with previous studies with follow-up that started at the time of CHD diagnosis or studies that only included an adult population. Another strength is that our study used registry-based data from a nationwide cohort that included all patients with CHD, and it was performed in a country with universal health coverage. Although the hospital registry has been nationwide since 1987, hospitals managing children with these conditions, particularly those providing heart surgery, have reported diagnoses since 1970. The hospitals have been mandated by law to register and deliver each diagnosis to the Ministry of Health and Welfare since 1964. Furthermore, each patient with CHD was matched by the year of birth, sex, and county with 10 controls from the general population. This provided additional strength to the comparison between patients and controls.

Our study has limitations. A major limitation of our study is that it was based only on administrative data. Therefore, clinical data or further information on radiation exposure were not available. Additionally, data from outpatient clinics before 2000 were not available. Another limitation is that we were not able to perform any validation of the CHD diagnosis. However, patients with CHD are generally managed in centers that specialize in CHD. Furthermore, external validation of other cardiovascular diseases in these registries has shown an overall positive predictive value of 85% to 95%,^26^ which is likely also applicable to misclassification of CHD.

## Conclusions

In conclusion, this study found that the overall risk of cancer in children and young adult patients with CHD in Sweden was more than twice that of healthy matched controls. The rates of cancer have increased in cancer rates of patients with CHD and, less strikingly, in controls between the 1970s and the early 1990s. Patients with complex heart lesions, such as conotruncal defects, had a particularly high risk of cancer. This finding suggests that particular attention should be paid to early warning signs of cancer and promotion of a healthy lifestyle. Further research on the mechanisms of cancer in this young patient group is warranted.

## Notes

Supplement. **eTable 1**. Congenital Heart Disease Diagnosis According to the *International Classification of Diseases and Related Health Problems* **eTable 2**. Cancer Diagnosis According to the *International Classification of Diseases and Related Health Problems* **eTable 3**. Incidence Rate of Cancer in Patients With Congenital Heart Disease and Controls According to Type of Cancer **eTable 4**. Incidence Rate of Cancer in Patients With Congenital Heart Disease and Controls According to Cancer Diagnosis **eTable 5**. Incidence Rate of Cancer in Patients With Congenital Heart Disease and Controls According to Age and Lesion Group **eFigure 1**. Cumulative Incidence of Cancer in Patients With Congenital Heart Disease and Controls According to Sex **eFigure 2**. Cumulative Incidence of Cancer in Patients With Congenital Heart Disease and Controls According to a Hierarchic Classification
