# Supplementary material for: Risk of Cancer Among Children and Young Adults With Congenital Heart Disease Compared With Healthy Controls
Source: JAMA Netw Open. 2019 Jul 5;2(7):e196762. doi: 10.1001/jamanetworkopen.2019.6762 (PMC12578487; doi:10.1001/jamanetworkopen.2019.6762)
Supplement: Supplement. — eTable 1. Congenital Heart Disease Diagnosis According to the International Classification of Diseases and Related Health Problems eTable 2. Cancer Diagnosis According to the International Classification of Diseases and Related Health Problems eTable 3. Incidence Rate of Cancer in Patients With Congenital Heart Disease and Controls According to Type of Cancer eTable 4. Incidence Rate of Cancer in Patients With Congenital Heart Disease and Controls According to Cancer Diagnosis eTable 5. Incidence Rate of Cancer in Patients With Congenital Heart Disease and Controls According to Age and Lesion Group eFigure 1. Cumulative Incidence of Cancer in Patients With Congenital Heart Disease and Controls According to Sex eFigure 2. Cumulative Incidence of Cancer in Patients With Congenital Heart Disease and Controls According to a Hierarchic Classification [file jamanetwopen-e196762-s001.pdf]

Supplementary Online Content

Mandalenakis Z, Karazisi C, Skoglund K, et al. Risk of cancer among children and young adults with congenital heart disease compared with healthy controls. *JAMA Netw Open*. 2019;2(7):e196762. doi:10.1001/jamanetworkopen.2019.6762

eTable 1. Congenital Heart Disease Diagnosis According to the *International Classification of Diseases and Related Health Problems*

eTable 2. Cancer Diagnosis According to the *International Classification of Diseases and Related Health Problems*

eTable 3. Incidence Rate of Cancer in Patients With Congenital Heart Disease and Controls According to Type of Cancer

eTable 4. Incidence Rate of Cancer in Patients With Congenital Heart Disease and Controls According to Cancer Diagnosis

eTable 5. Incidence Rate of Cancer in Patients With Congenital Heart Disease and Controls According to Age and Lesion Group

eFigure 1. Cumulative Incidence of Cancer in Patients With Congenital Heart Disease and Controls According to Sex

eFigure 2. Cumulative Incidence of Cancer in Patients With Congenital Heart Disease and Controls According to a Hierarchic Classification

This supplementary material has been provided by the authors to give readers additional information about their work.

© 2019 Mandalenakis Z et al. *JAMA Network Open.*

eTable 1. Congenital Heart Disease Diagnosis According to the *International Classification of Diseases and Related Health Problems*

Diagnosis	ICD-8	ICD-9*	ICD-10
Tetralogy of Fallot	746.29	745C	Q21.3
Transposition of the great vessels	746.1	745B	Q20.3
Common arterial trunk	746.0	745A	Q20.0
Ventricular septal defect	746.39	745E	Q21.0
Atrial septal defect or patent foramen ovale	746.4	745F	Q21.1
Congenital tricuspid stenosis or atresia	746.54	746B	Q22.4
Ebstein's anomaly	746.54	746C	Q22.5
Congenital stenosis of the aortic valve	746.73	746D	Q23.0
Congenital insufficiency of the aortic valve	746.79	746E	Q23.1
Congenital mitral stenosis	746.59	746F	Q23.2
Congenital mitral insufficiency	746.59	746G	Q23.3
Hypoplastic left heart syndrome	746.74	746H	Q23.4
Congenital subaortic stenosis	746.79	746W	Q24.4
Cor triatriatum	746.82	746W	Q24.2
Infundibular pulmonic stenosis	746.63	746W	Q24.3
Congenital coronary vessel anomalies	746.85	746W	Q24.5

© 2019 Mandalenakis Z et al. *JAMA Network Open.*

Congenital heart block	746.86	746W	Q24.6
Coarctation of the aorta	747.19	747B	Q25.1
Interruption of the aortic arch<br><br>(atresia or stenosis of the aorta)	747.19	747B	Q25.2, Q25.3
Other unspecified congenital malformations of the aorta	747.29	747C	Q25.4, Q25.8,<br>Q25.9
Congenital malformations of the pulmonary artery	747.34,<br>747.39	747D	Q25.5–Q25.7
Congenital malformations of the great veins	747.49,<br>747.59	747E	Q26
Cor biloculare	746.89	745H	Q20.8
Double outlet right ventricle	746.19	745B	Q20.1
Double outlet left ventricle	746.19	745B	Q20.2
Double inlet ventricle	746.37	745D	Q20.4
Discordant atrioventricular connection	746.19	745B	Q20.5
Isomerism of atrial appendages	745.89	745W	Q20.6
Unspecified congenital malformations of the cardiac chambers	746.89	746X	Q20.8, Q20.9
Atrioventricular septal defect	746.47	745G	Q21.2
Aortopulmonary septum defect	746.09	745W	Q21.4
Other congenital malformations of the cardiac septum	745.89	745W	Q21.8
Unspecified congenital malformations of the cardiac septum	745.99	745X	Q21.9

Pulmonary valve atresia	746.64	746A	Q22.0
Congenital stenosis of the pulmonary valve	746.63	746A	Q22.1
Congenital pulmonary valve insufficiency	746.69	746A	Q22.2
Other congenital malformations of the pulmonary valve	746.00	746A	Q22.3
Hypoplastic right heart syndrome	746.69	746B	Q22.6
Other congenital malformations of the tricuspid valve	746.54	746B	Q22.8, Q22.9
Other congenital malformations of aortic and mitral valves	746.89	746W	Q23.8, Q23.9
Congenital phlebectasia	747.89	747G	Q27.4
Other specified congenital malformations of the heart	746.89	746W	Q24.8
Unspecified congenital malformations of the heart	746.84	746X	Q24.9
Patent ductus arteriosus	747.0	747A	Q25.0
Unspecified congenital malformations of the circulation	747.9	747X	Q28.9
Sequestration of the lungs	748.5	748F	Q33.2
Secondary hypertension	405	405	I15.8, I15.9
Vitium organicum cordis (VOC)	-	-	I33–37
ICD, International Classification of Diseases. *Swedish version of the ICD-9.			

© 2019 Mandalenakis Z et al. *JAMA Network Open.*

eTable 2. Cancer Diagnosis According to the *International Classification of Diseases and Related Health Problems*

Diagnosis	ICD-8	ICD-9*	ICD-10
Malignant neoplasms of lip, oral cavity and pharynx	140-149	140-149	C00-C14
Malignant neoplasms of digestive organs	150-159	150-159	C15-C26
Malignant neoplasms of respiratory and intrathoracic organs	160-163	160-165	C30-C39
Malignant neoplasms of bone and articular cartilage	170-171	170	C40-C41
Melanoma and other malignant neoplasms of skin	172-173	172-173	C43-C44
Malignant neoplasms of mesothelial and soft tissue	-	158,162,163,171,173	C45-C49
Malignant neoplasms of breast	174	174-175	C50
Malignant neoplasms of female genital organs	180-184	179-184,236C	C51-C58
Malignant neoplasms of male genital organs	185-187	185-187	C60-C63
Malignant neoplasms of urinary tract	188-189	188-189	C64-C68
Malignant neoplasms of eye, brain and other parts of central nervous system	190-192	190-192	C69-C72
Malignant neoplasms of thyroid and other endocrine glands	193-194	193-194	C73-C75
Malignant neoplasms o ill-defined, secondary and unspecified sites	195-198	195-199	C76-C80
Malignant neoplasms, stated or presumed to be primary, of lymphoid, haematopoietic and related tissue	200-209	200-209	C81-C96

© 2019 Mandalenakis Z et al. *JAMA Network Open.*

Malignant neoplasms of independent (primary) multiple sites	199	199	C97
ICD, International Classification of Diseases. *Swedish version of the ICD-9.			

eTable 3. Incidence Rate of Cancer in Patients With Congenital Heart Disease and Controls According to Type of Cancer

Cancer type		Events<br>Cases/controls	Incidence rate of cancer<br>(cases/controls)*	Incidence rate-ratio for cancer<br>(95% CI)
Carcinoma		125/603	2.11/0.96	2.25 (1.92-2.73)
Sarcoma		65/304	1.10/0.48	2.27 (1.71-2.96)
Lymphoma, Leukemia		127/461	2.14/0.74	2.93 (2.39-3.57)
Other types		196/1176	3.31/1.88	1.81 (1.57-2.11)

* Incidence rate per 10,000 person-years

© 2019 Mandalenakis Z et al. *JAMA Network Open.*

eTable 4. Incidence Rate of Cancer in Patients With Congenital Heart Disease and Controls According to Cancer Diagnosis

Cancer diagnosis	Events cases/controls (n)	Incidence rate of cancer (cases/controls)*	Incidence rate-ratio for cancer (95% CI)
Malignant neoplasms of lip, oral cavity and pharynx	23/80	0.40/0.10	3.08 (1.92-4.90)
Malignant neoplasms of digestive organs	47/142	0.80/0.20	3.58 (2.58-4.98)
Malignant neoplasms of respiratory and intrathoracic organs	13/67	0.20/0.10	2.10 (1.24-3.80)
Malignant neoplasms of bone and articular cartilage	14/75	0.22/0.14	1.99 (1.13-3.53)
Melanoma and other malignant neoplasms of skin	57/434	1.02/0.70	1.44 (1.09-1.90)
Malignant neoplasms of mesothelial and soft tissue	20/99	0.32/0.20	2.16 (1.27-3.50)
Malignant neoplasms of breast	22/134	0.42/0.19	1.81 (1.24-2.83)
Malignant neoplasms of female genital organs	25/134	0.40/0.23	2.10 (1.45-3.22)
Malignant neoplasms of male genital organs	29/208	0.54/0.30	1.51 (1.06-2.23)
Malignant neoplasms of urinary tract	18/70	0.30/0.10	2.76 (1.63-4.64)
Malignant neoplasms of eye, brain and other parts of central nervous system	52/236	0.93/0.42	2.33 (1.72-3.15)

© 2019 Mandalenakis Z et al. *JAMA Network Open.*

Malignant neoplasms of thyroid and other endocrine glands	15/115	0.29/0.18	1.40 (0.78-2.41)
Malignant neoplasms of ill-defined, secondary and unspecified sites	61/271	0.98/0.37	2.44 (1.90-3.23)
Malignant neoplasms, stated or presumed to be primary, of lymphoid, haematopoietic and related tissue	127/461	2.11/0.72	2.93 (2.41-3.57)
Malignant neoplasms of independent (primary) multiple sites	0/1	0/0	-

* Incidence rate per 10,000 person-years

© 2019 Mandalenakis Z et al. *JAMA Network Open.*

eTable 5. Incidence Rate of Cancer in Patients With Congenital Heart Disease and Controls According to Age and Lesion Group

Lesion group	Incidence rate of cancer by age (case/control)*			
	0 – 4	5 – 9	10 – 17	18 – 42
Conotruncal defects	395.65 / 68.65	42.62 / 54.26	138.81 / 76.97	592.45 / 422.14
Severe nonconotruncal defects	301.15 / 80.71	80.74 / 26.93	260.19 / 58.97	434.64 / 514.13
Coartation of aorta	57.07 / 39.17	57.75 / 33.59	109.38 / 70.08	588.08 / 426.84
Ventricular septum defect	324.95 / 42.49	121.39 / 46.87	131.12 / 75.43	691.67 / 493.77
Atrial septum defect	247.32 / 97.28	125.03 / 27.39	157.08 / 74.28	667.02 / 521.12
Other heart and circulatory system anomalies	267.97 / 64.35	180.14 / 44.75	187.43 / 44.11	617.22 / 452.03

* Incidence rate per 10,000 person-years

© 2019 Mandalenakis Z et al. *JAMA Network Open.*

eFigure 1. Cumulative Incidence of Cancer in Patients With Congenital Heart Disease and Controls According to Sex

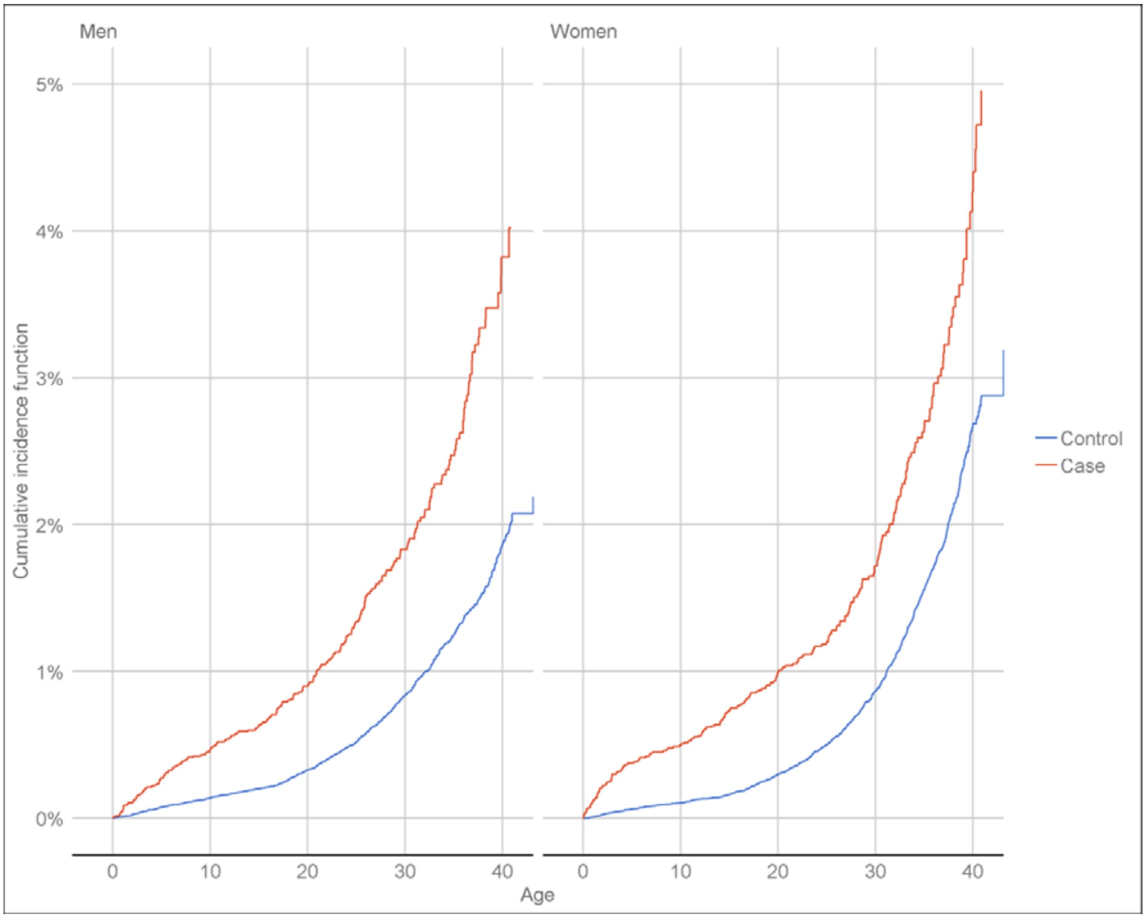

© 2019 Mandalenakis Z et al. *JAMA Network Open.*

eFigure 2. Cumulative Incidence of Cancer in Patients With Congenital Heart Disease and Controls According to a Hierarchic Classification

Lesion group 1 was defined as patients with conotruncal defects such as common arterial trunk, transposition of the great vessels, tetralogy of Fallot, or aortopulmonary septum defect. Lesion group 2 was defined as patients with non-conotruncal defects such as endocardial cushion defects, common ventricle and hypoplastic left heart syndrome. Lesion group 3 was defined as patients with coartation of the aorta. Lesion group 4 was defined as patients with ventricular septal defect. Lesion group 5 was defined as patients with atrial septal defect. Legion group 6 included all other heart and circulatory system anomalies and all other CHD diagnosis that were not included in the other five lesion groups

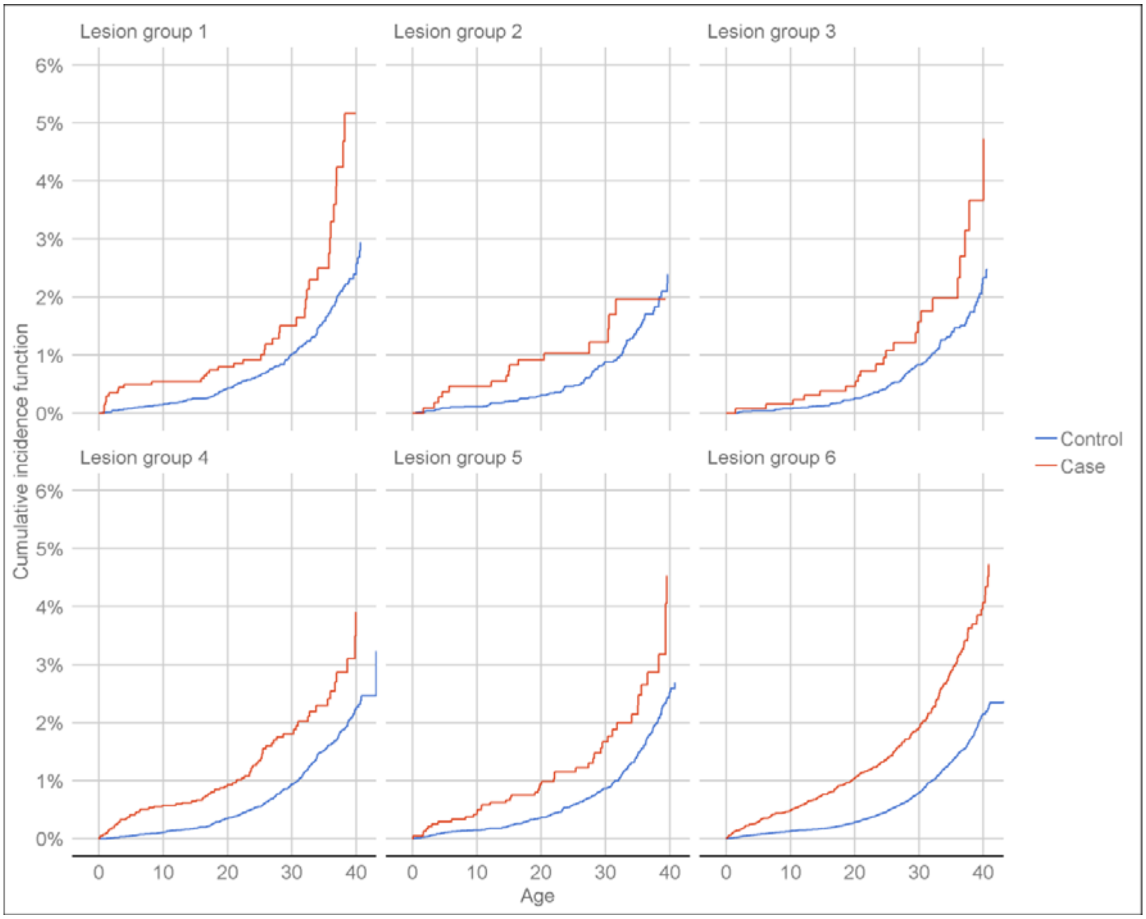

© 2019 Mandalenakis Z et al. *JAMA Network Open.*
